# Splenic Injury: A Curious Complication of Colonoscopy

**DOI:** 10.7759/cureus.36724

**Published:** 2023-03-27

**Authors:** Zoilo K Suarez, Oscar L Hernandez, Pedro J Diaz, Samantha Matott, Quan Ta

**Affiliations:** 1 Internal Medicine, Florida Atlantic University Charles E. Schmidt College of Medicine, Boca Raton, USA; 2 Internal Medicine, University of Texas Health Science Center at San Antonio, San Antonio, USA; 3 Medicine, Florida Atlantic University Charles E. Schmidt College of Medicine, Boca Raton, USA

**Keywords:** colonoscopy complications, spleen, splenic injury, splenic hematoma, splenic laceration

## Abstract

Colonoscopies are generally considered a safe procedure with an overall complication rate of 0.5%. Splenic injuries, including laceration, subcapsular hematoma, and rupture, have been thought to be underreported in the currently available literature. The etiology of splenic injury remains unknown, although excessive splenocolic ligament stretching and traction of adhesions have been hypothesized to play a role in its development. Even though conservative, percutaneous, and surgical strategies have been described in the literature, these strategies have been associated with higher mortality, and there is no consensus on the optimal approach to management. We present the case of a patient who sustained a splenic injury after a colonoscopy and was successfully managed with conservative measures.

## Introduction

Colonoscopies have become a routine medical procedure, recommended for most adults over 45 years old in the United States [1]. Splenic laceration [2], subcapsular hematoma [3], and complete splenic rupture [4], while uncommon [5], have been reported in the literature and associated with high morbidity [6]. The most commonly reported symptom for patients experiencing these complications is abdominal pain which may be non-specific and not contained to the left upper quadrant. Despite the various types of splenic injury, there is no specific consensus on the optimal approach toward the management of this complication. We present the case of a patient who had a splenic injury after a colonoscopy and was successfully managed conservatively.

## Case presentation

A 79-year-old female with a history of cholecystitis requiring laparoscopic cholecystectomy, long-standing gastroesophageal reflux disease, hypertension, and hypothyroidism was evaluated at her gastroenterologist’s office for a routine upper endoscopy and colonoscopy. While in the recovery room, around 30 minutes following the procedure, she complained of diffuse abdominal discomfort. Her symptoms were initially suspected to be secondary to excessive air remaining in the digestive tract; therefore, intraluminal air decompression introducing the scope up to the hepatic flexure was performed. Following the second procedure, the patient developed worsening diffuse abdominal pain for which she was immediately instructed to report to our emergency department (ED) for further evaluation.

**Figure 1:**
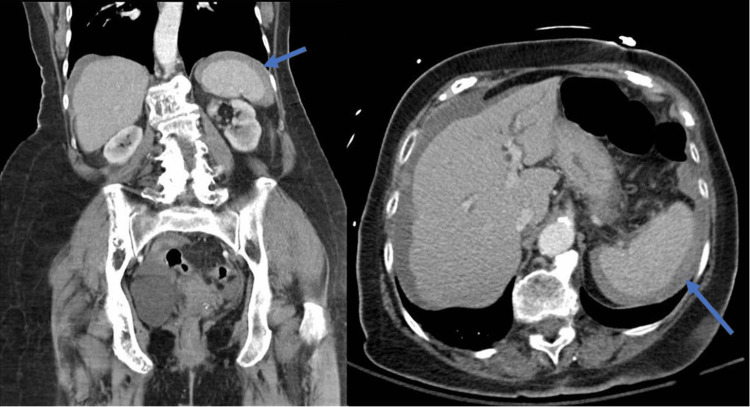
Computerized tomography (CT scan) of the abdomen and pelvis CT scan of the abdomen and pelvis showed moderate ascites and complex components at the left paracolic gutter and margins of the spleen suggestive of hemoperitoneum. A small hiatal hernia, an intrahepatic biliary duct with common bile duct dilation measuring 1.9 cm, and a 1.5 cm cystic lesion at the pancreatic body were also noted.

In the ED, the patient was found to be tachycardic and tachypneic. She reported sharp abdominal pain in the periumbilical area radiating to her epigastrium and left upper quadrant, which worsened upon taking a deep breath and improved when lying still. Her symptoms were associated with chills and nausea without emesis. Moderate abdominal tenderness to superficial and deep palpation, primarily in her left upper quadrant, was noted on the physical exam without guarding or the presence of peritoneal signs. Laboratory studies showed a hemoglobin of 12 g/dL. A computerized tomography (CT scan) of the abdomen with intravenous and oral contrast revealed moderate ascites and complex components at the left paracolic gutter and margins of the spleen suggestive of hemoperitoneum (Figure 1). A small hiatal hernia, an intrahepatic biliary duct with common bile duct dilation measuring 1.9 cm, and a 1.5 cm cystic lesion at the pancreatic body were also noted. Due to the presence of significant abdominal pain, and the previously mentioned imaging findings, general surgery, and gastroenterology services were consulted, and the patient was admitted to the hospital.

Given the lack of peritoneal signs, the decision was made to manage conservatively with nil per oral (NPO), intravenous fluids, pain control, and serial hemoglobin and hematocrit (H&H) measurements. During her hospital stay, her hemoglobin slowly declined to 10.0 g/dL, but she did not require any blood transfusions. Ultimately, the patient had a gradual resolution of her symptoms, and the patient was able to tolerate diet, following which she was discharged after four days and recommended to follow up with her gastroenterologist and primary care physician as an outpatient. On follow-up six months after this intervention, the patient remained asymptomatic and did not require any further hospitalizations.

## Discussion

Colonoscopies are generally considered a safe procedure with an overall complication rate of 0.5%, including bowel perforation, bleeding, and less commonly, splenic injury [5]. Splenic injuries, including laceration, subcapsular hematoma, and rupture, have a reported incidence rate of 0.0045% but have been thought to be underreported in the literature [6,7]. Risk factors such as female sex, older age, multiple abdominal surgical procedures, comorbidities, anticoagulation, less experienced endoscopists, and polypectomy have been associated with this complication [8,9]. Although excessive splenocolic ligament stretching and traction of adhesions have been hypothesized to play a role in the pathogenesis, the exact mechanism remains to be elucidated. It has been associated with higher mortality and ICU length of stay [10].

A splenic injury commonly presents with abdominal pain radiating to the left shoulder within 24 hours of the procedure, even though it has been reported up to 10 days after. Other signs and symptoms can include abdominal distention, peritonitis, and hemodynamic instability [11,12]. Diagnostic modalities for suspected splenic injuries can range from a focused assessment with sonography (FAST) to the gold standard of computerized tomography of the abdomen and pelvis with intravenous contrast, which can grade the lesion.

Conservative [13], percutaneous [14], and surgical strategies have been described in the literature [15]. Patients who are hemodynamically stable are generally managed conservatively with parenteral fluids, pain control, and serial monitoring of H&Hs, as in our case. However, more invasive strategies such as arterial embolization or splenectomy are required for unstable patients. Subsequently, patients who undergo splenectomy should be vaccinated against encapsulated organisms.

Finally, colonoscopy is generally regarded as a safe procedure with a low risk of complications. Given the recently updated guidelines [1,16] for screening colonoscopies in the United States, it is important to increase awareness of this complication in order to provide the best possible care for our patients.

## Conclusions

Splenic injury is a rare and potentially underreported and underdiagnosed complication of colonoscopy that could have severe consequences; therefore should be considered in any patient who develops abdominal pain after a colonoscopy. Patients with multiple risk factors should be placed in the left lateral position to facilitate the colonoscope insertion and prevent this complication. Early suspicion of a splenic injury can lead to more prompt recognition of this complication and expeditious utilization of interventions to improve morbidity and mortality in these patients. We consider the decision of conservative versus invasive management could rely on the clinical presentation of the patient, including the presence or lack of peritoneal signs, blood pressure, hemoglobin levels, evidence of vascular injury in imaging, and grade of the splenic injury per the American Association for the Surgery of Trauma-Organ Injury Scale (AAST-OIS), but larger studies are needed. Evaluation by a multidisciplinary team, including a gastroenterologist, an internist, and a general surgeon, is the cornerstone of comprehensive care.

## References

[1] Shaukat A, Kahi CJ, Burke CA, Rabeneck L, Sauer BG, Rex DK (2021). ACG Clinical Guidelines: colorectal cancer screening 2021. Am J Gastroenterol.

[2] Rafa O, Campeas A, Basile E (2021). A case report of colonoscopy-induced splenic laceration: risks and outcomes. Am J Med Case Rep.

[3] Enofe I, Burch J, Yam J, Rai M (2020). Iatrogenic severe splenic injury after colonoscopy. Case Rep Gastrointest Med.

[4] González-Andrades E (2021). Splenic rupture secondary of colonoscopy. Med Clin (Barc).

[5] Cathala Esberard B, Mohseni M (2020). Splenic injury: a rare complication of lower endoscopy. BMJ Case Rep.

[6] Ullah W, Rashid MU, Mehmood A, Zafar Y, Hussain I, Sarvepalli D, Hasan MK (2020). Splenic injuries secondary to colonoscopy: rare but serious complication. World J Gastrointest Surg.

[7] Díaz Alcázar MD, García Robles A, Martín-Lagos Maldonado A (2021). Splenic rupture as an endoscopic complication: as rare as it appears?. Rev Esp Enferm Dig.

[8] Denis B, Gendre I, Weber S, Perrin P (2021). Adverse events of colonoscopy in a colorectal cancer screening program with fecal immunochemical testing: a population-based observational study. Endosc Int Open.

[9] Olaiya B, Adler DG (2020). Adverse events after inpatient colonoscopy in octogenarians. J Clin Gastroenterol.

[10] Bielawska B, Hookey LC, Sutradhar R (2018). Anesthesia assistance in outpatient colonoscopy and risk of aspiration pneumonia, bowel perforation, and splenic injury. Gastroenterology.

[11] Abunnaja S, Panait L, Palesty JA, Macaron S (2012). Laparoscopic splenectomy for traumatic splenic injury after screening colonoscopy. Case Rep Gastroenterol.

[12] Masood DE, Strauss P (2023). Case report on severe splenic injury following colonoscopy with disproportionately stable presentation: a rural hospital perspective. Int J Surg Case Rep.

[13] Ungprasert P, Jaruvongvanich V (2018). Splenic injury: an unusual complication of colonoscopy. Chin Med J (Engl).

[14] Wright JB, Gray S, Huynh D (2021). A case of iatrogenic splenic injury following routine colonoscopy with possible influence of unique anatomy due to severe scoliosis. Cureus.

[15] Patel DD, Shih-Della Penna DC, Terry SM (2020). Splenic trauma from colonoscopy: a case series. Int J Surg Case Rep.

[16] US Preventive Services Taskforce (2023). Colorectal cancer: screening. https://uspreventiveservicestaskforce.org/uspstf/recommendation/colorectal-cancer-screening#fullrecommendationstart.

